# Reports of work-related traumatic events: A mixedmethods study

**DOI:** 10.18332/ejm/100611

**Published:** 2018-12-31

**Authors:** Yvonne Fontein-Kuipers, Hester Duivis, Verena Schamper, Veerle Schmitz, Anouk Stam, Diana Koster

**Affiliations:** 1 Rotterdam University of Applied Sciences, Rotterdam, the Netherlands; 2 Antwerp University, Belgium; 3 AMC Academic Medical Centre, University of Amsterdam, Amsterdam, the Netherlands; 4 Women’s coaching practice

**Keywords:** midwifery, mixed-methods study, second victim, secondary traumatic stress, work-related traumatic event

## Abstract

**INTRODUCTION:**

There is limited evidence of the effect and impact on midwives of being involved or witnessing traumatic work-related events. We categorised midwives’ selfreported traumatic work-related events and responses to an event and explored the impact on the midwives’ professional and personal life.

**METHODS:**

A sequential explanatory mixed-methods study, consisting of a questionnaire and semi-structured interviews for midwives who practised or who had practised in the Netherlands or Flanders.

**RESULTS:**

In total, 106 questionnaires were completed. We categorised various workrelated traumatic events: witnessing birth trauma/complications (34%), death (28.3%), (mis)management of care (19.8%), events related to the perceived social norm of maternity services’ practitioners (9.5%), events related to environmental and contextual issues (5.6%) and to (mis)communication (2.8%). Sharing the experience with colleagues, family and friends, a supervisor or the woman involved in the event, was the most common response. In all, 74.5% of the participants still experienced the influence of work-related events in day-to-day practice and 37.5% still experienced the effects in their personal life. The scores of three participants (3.2%) indicated the likelihood of post-traumatic stress. Twenty-four interviews were conducted. Four themes emerged from the content analysis: 1) Timeline, 2) Drawing up the balance of relations with others, 3) Fretting and worrying, and 4) Lessons learned.

**CONCLUSIONS:**

Various work-related traumatic events can impact on midwives’ professional and/or personal life. Although not all midwives reported experiencing (lasting) effects of the events, the impact was sometimes far-reaching. Therefore, midwives’ experiences and impact of work-related traumatic events cannot be ignored in midwifery practice, education and in supervision or mentoring.

## INTRODUCTION

Midwifery work is emotionally challenging as midwives are part of one of the most important life-events of a woman: giving birth and becoming a mother; sharing moments of joy; but also being involved in loss and/or trauma. In addition, the midwife carries responsibility for the safety and wellbeing of mother and child^1^, constantly weighing and considering different factors that may cause inter and extra-personal conflicts^2^. The very nature of a midwife’s role, i.e. to be *with woman*, may put midwives in a vulnerable position of exposure to upsetting and distressing events while caring for childbearing women. Close involvement with women and personal emotional investment in the caring relationship, an aspect of the woman-midwife relationship^3^, can lead to secondary traumatic stress^4-6^. Midwives are regarded as second victims when they are involved in an adverse event, a medical error and/or injury involving the childbearing woman and/or the (unborn) child and when they are traumatised by the event^7^. The midwifery profession in the Netherlands and Flanders is under increasing strain from a rising number of maternal reports of traumatic childbirth experiences^8,9^ — experiences that most likely affect midwives when witnessing and/ or sharing the experiences of women or being involved in management leading to trauma^7^. Thirteen per cent of practising midwives in the Netherlands reported to having been involved in a traumatic work-related event, resulting in post-traumatic stress for some of them (2.2% of practising midwives)^10^. Studies among midwifery students reported high levels of involvement in traumatic work-related events^11-13^.

There is substantial reporting of being involved in or witnessing traumatic events in midwifery^4-6,14-18^. However, there is very limited research concerning the possible effects and impact on midwives of being involved or witnessing traumatic work-related events — on a professional as well as on a personal level. This study aimed to investigate accounts of midwives about work-related events, being experienced as traumatic, in order to categorize: i) the types of events, ii) midwives’ responses to the events, and iii) to explore their experiences of the aftermath of the event on professional and personal life.

## METHODS

We conducted a sequential explanatory mixed-methods design that was quantitatively-driven (QUAN→qual)^19-21^. The study consisted of two phases: 1) a questionnaire, and 2) interviews. In our case we connected the findings of Phase One to the data collection in Phase Two^20,22^. Qualified midwives in the Netherlands and Flanders currently practising or who had been practising as a midwife (full or part-time), with Dutch linguistic proficiency, were eligible for both phases of the study. We did not apply limits to the years of recent work experience or on how long ago the event had taken place. No definition or criteria for a traumatic work-related event were provided — as this was self-defined by the participants based on their own judgement of their personal experiences^23^. We aimed to recruit midwives from various regions and working in various care settings, including the primary (community) and hospital setting.

### Phase One

A self-completed questionnaire was developed for the purpose of the study with the aim to categorise the selfreported traumatic work-related events and midwives’ responses to the events. Alongside sociodemographic and personal details, the questionnaire included: i) three items to describe the traumatic event (maximum of 1000 words) and the influences on professional and personal life (maximum of 500 words), ii) one item categorising responses to the event, iii) two items with a 5-point rating scale (with the extremes labelled ‘not at all’ to ‘all the time’) to measure the influence on professional and personal life, and iv) five items measuring stress with a 5-point rating scale (with the extremes labelled ‘not at all’ to ‘very much/very often’). Three or more scores of ≥4 were considered clinically relevant for post-traumatic stress (PTSD). The five items we used in this study stem from the DSM-IV PTSD categorisation^24^, designed and used by one of the authors (DK) for in-person assessment of PTSD. Participants in our study were asked: *‘describe a work-related event you have experienced as very upsetting, very distressing or traumatic’*. Participants were instructed that it did not matter how long ago the event had happened — most important was that it (still) mattered to them. The item that measured responses to the event were derived from the literature and professional counselling expertise (HD, DK). To ensure validity, the complete questionnaire was pre-tested among 35 student midwives that resulted in the rephrasing of one ambiguous question, revisions in wording, and allowing 1000 words to describe the traumatic event (see Supplementary file of questionnaire for items i–iv).

To recruit participants, we sent a total of 241 invitation emails to midwifery community practices and obstetric units in the South–West of the Netherlands and in Mid– North Flanders that offer clinical placements to midwifery students. The link to the online questionnaire was included in the email. Data were collected between September 2016 and March 2017 with the online questionnaire tool Survio. We categorised the open answers, using an inductive content analysis producing a summary of categories^25^, representing types of traumatic events and midwives’ response strategies. The categories were quantified. We used SPSS version 23.0 for our analyses.

### Phase Two

We performed a qualitative descriptive study to seek elaboration, illustration and clarification of the Phase One findings^19-21^. The focus was on the impact of the aftermath of the event on the professional and personal life of midwives. A topic list (Box 1) was constructed based on the Phase One findings. We conducted semi-structured interviews from April to July 2017, giving voice to the perceived meaning of these experiences. To recruit participants, we had asked midwives in Phase One if they would be interested in being interviewed^20^. Twelve midwives responded positively. As we aimed to include more participants, we posted a recruiting message on a Dutch and a Flemish midwifery Facebook group, allowing snowballing^25^. Fifteen midwives showed interest in the study. One midwife withdrew, two interviews could not be scheduled due to being on-call, after which 24 midwives remained. We performed a pilot-interview with two midwives to increase reliability and internal consistency of the usage of the topic list^26^.

**Box 1. Topic list**

Nature of the incident

Initial response/emotions

Short-term coping strategies and processing

Longer-term coping strategies and processing

Current recall/memory

Effects/impact on professional life — management of care/professional behaviour, support, job satisfaction Effects/impact on personal life — relations/social environment, (daily) activities, quality of life, support, health and wellbeing

View/perceptions on midwifery as a profession

Interviews were audio recorded and transcribed verbatim. We used a process of open coding, creating categories and abstraction, known as content analysis^27^. We collected the labels, clustered them in preliminary categories and ordered similar categories into core themes^25^. We reached theoretical saturation on all categories. The researchers interpreted the findings, discussed them and mutually agreed on the identified themes.

### Ethical consideration

The study was conducted in accordance with the Helsinki declaration, and participation was voluntary. Because of the non-interventionsist character of the study, ethical approval was not required according to Dutch ethical research standards (reference WC2016–055; http://www.ccmo.nl/en/your-research-does-it-fall-under-the-wmo). Participation in the survey was regarded as consent. At the time of the interviews the presence of the participants was a confirmation of their consent to participate. Participation was anonymous. Prior to the interviews, consent for audio recording of the interviews was obtained. The transcribed data were anonymised. Information was given that only the researchers had access to the data, and that findings would be published without identifiable information on the participants. One of the authors (DK) was a certified counsellor who was available to participants for whom reporting or discussing the traumatic events precipitated/ reactivated distress, requiring support.

## RESULTS

### Phase One: Survey

We received 134 questionnaires of which 106 completed questionnaires could be included in the analysis (79%). When the description of the traumatic event was missing, we excluded the questionnaire for analysis. The participants showed a variety in years of age (23 to 63) and years of work experience (1 to 38). Most participants worked or had worked in the Netherlands (84.9%), predominantly in primary care (community) settings (70.8%). More than half of the participants held a Bachelor degree (55.6%) (Table 1). The events that were described by the participants had happened between 1 and 37 years ago. The participants provided rich data that were coded and quantified.

**Table 1:** Characteristics of midwives Phase One (N=106)

*Characteristics*	*Mean±SD (range)*
Age (years)	40.4±11.07 (23–63)
Years of work experience	13.8±9.42 (1–38)
	***n (%)***
Midwife in the Netherlands	90 (84.9)
Midwife in Belgium (Flanders)	16 (15.1)
Diploma	25 (23.6)
Bachelor degree	59 (55.6)
Master degree	22 (20.8)
Working in primary care setting	75 (70.8)
Working in hospital setting	31 (29.2)

All participants provided narratives of an upsetting, distressing or traumatic work-related event, which were coded based on the character of the event. All participants clearly described the event as well as the context of the situation. The events involved witnessing obstetric trauma/ complications (34%), but also related to death of women and (unborn) children (28.3%) and (mis)management of care (19.8%). Events were related to the perceived social norm of maternity services’ practitioners (9.5%), to environmental and contextual issues (5.6%) and to suboptimal communication (2.8%) (Table 2).

**Table 2:** Types of work-related traumatic events (N=106)

*Categories*	*n (%)*
(Intra-uterine) death	28 (26.4)
Resuscitation — including maternal resuscitation, n=2	11 (10.4)
Shoulder dystocia	9 (8.5)
Suboptimal — (mis) management of care	8 (7.5)
Severe maternal morbidity	7 (6.6)
Lack of collegiality — including bullying	6 (5.7)
Delayed assistance (help)	6 (5.7)
Postpartum haemorrhage	4 (3.8)
Midwife’s care (management) being questioned by other practitioners	4 (3.8)
Risky — unsafe management of care (own and other’s)	4 (3.8)
Witnessing very invasive (inhumane) ways of performing interventions	3 (2.8)
Insolvable maternal despair — including maternal inability to cope emotionally	3 (2.8)
Involvement in legislation	3 (2.8)
Suboptimal communication with hospital staff	3 (2.8)
Maternal death	2 (1.9)
Woman with a complicated medical history combined with negative birth outcome	2 (1.9)
Unsafe environment — including violence (aggression)	2 (1.9)
Being pregnant combined with involvement in neonatal mortality	1 (0.9)

The participants self-identified their responses to their described traumatic event. Self-disclosure by means of debriefing and discussion with either colleagues, family and friends, supervisor or the woman involved in the event, were the most reported response strategies. Avoidance was another identified response strategy (do nothing, trivialise) as well as help-seeking (professional help, including general practitioner). More conclusive response strategies were discontinuation of practice, including sick leave (Table 3).

**Table 3:** Responses to work-related traumatic events

*Strategies*	*n (%)*
Discussed with colleagues	87 (82.1)
Talked to family and friends	64 (60.4)
Discussed with the woman (partner)	54 (57.2)
Written it down (diary)	16 (17.0)
Nothing	13 (13.8)
Trivialising the situation	13 (13.8)
Talked to supervisor	11 (11.7)
Discontinued practice	8 (8.5)
Professional help	4 (4.2)
Discussed with General Practitioner	1 (1.1)
Filed a complaint	1 (1.1)
Sick leave	1 (1.1)

The influence experienced on day-to-day practice showed a mean ± SD score of 2.2±0.96 (range 1–5). The participants described if and how they were still aware of the influence of the described upsetting, distressing or traumatic work-related event on work floor level, their dayto-day practice. A quarter of the respondents reported to experience no after-effects of the event. Three-quarters reported positive and negative experiences as a result of the event. Positive influences were identified as professional development or taking more time for women. However, most responses had a rather negative character, represented in reports of being less at ease and reduced happiness at work, including reduced work satisfaction. The events also resulted in change of care management, anticipating worstcase scenarios. Leaving midwifery was also reported (Table 4).

**Table 4:** Influence of the event on day-to-day practice

*Categories*	*n (%)*
No influence on daily practice	27 (25.5)
Being more fearful on a daily basis	22 (23.3)
Feeling apprehensive in a similar situation	19 (20.2)
More proactive decisions interventions (referral)	19 (20.2)
Feeling more insecure on a daily basis	15 (16.0)
Change of work — career (position)	12 (11.7)
Learning curve: ‘chalk it up as experience’	11 (11.6)
Flashbacks in similar situations	7 (7.4)
Taking more time for women	5 (5.5)
Disturbed collaborative (integral) practice	5 (5.3)
Loss of job satisfaction	4 (4.2)
Burn-out	2 (2.1)
Lack of concentration	2 (2.1)
Use of communication tools (e.g. SBAR)	2 (2.1)
Feeling physically ill	2 (2.1)
Not going to work	1 (1.1)

The influence experienced on a personal level showed a mean score of 1.77±1.12 (range 1–5). The participants described if and how they were still aware of the influence of the described upsetting, distressing or traumatic workrelated event on their daily personal life, allowing multiple answers. More than half of the number of participants reported to experience no influence on their personal life resulting from the event. Again, positive and negative experiences as a result of the event were reported. Personal growth was regarded to be a positive effect, while emotional and psychosomatic problems and substance abuse were negative effects (Table 5).

**Table 5:** Influence of the event on personal life

*Categories*	*n (%)*
No influence on personal life	59 (62.5)
Lack of interest in others	20 (21.2)
Personal growth after incident	17 (18.0)
Being easily frustrated (irritated)	10 (10.6)
Being more fearful than before	9 (9.5)
Having trouble sleeping (insomnia)	8 (8.5)
Psychosomatic complaints	4 (4.2)
Inactivity (being socially inactive)	4 (4.2)
Start smoking (and/or drinking)	2 (2.1)

The five items measuring stress and/or post-traumatic stress showed low scores; fear/horror/panic scoring the highest and avoidance the lowest (Table 6). The items showed acceptable internal consistency (α=0.76) and acceptable inter-item correlation (0.35). Three participants (3.2%) had three or more scores ≥4, indicating that these participants were likely to meet the threshold requirements for PTSD.

**Table 6:** Measurement of PTSD

*Items*	*Symptom PTSD*	*Mean±SD (range)*
Does the event still play a distressing role in your life and currently affect your professional, personal or social life?	Distress	1.74±1.02 (1–5)
Does the event still cause intense fear, panic or helplessness?	Fear/horror/panic	2.01±0.97 (1–5)
Do you currently experience any of these signs/symptoms: irritability, outbursts of anger, lack of concentration, difficulty sleeping, palpitations and/or (excessive) sweating when you think back to what happened?	Trauma-related arousal and reactivity	1.36±0.77 (1–5)
Do you still have upsetting thoughts, memories, dreams, flashbacks or images that replay what happened?	Re-experience	1.33±0.74 (1–4)
Are you deliberately trying not to think about what happened, to avoid places or people related to the event because you feel upset by reminders of the event?	Avoidance	1.16±0.56 (1–4)

### Phase Two: Interviews

We conducted 24 individual in-depth interviews with practising community midwives and midwives who had discontinued their practice, from various regions in the Netherlands. The participants were between 30 and 51 years of age and had between 1 to 22 years of work experience.

No Flemish midwives were included, due to non-response to recruitment messages. Events included the management of obstetric complications, intra-uterine and neonatal death, resuscitation and litigation. Participants referred to events that had happened up to fifteen years ago. Twenty of the 24 midwives had considered discontinuing practice, of which five midwives had discontinued practice as a direct or indirect result of the event. Six midwives had sought professional support after the event. The experiences of the aftermath of the event were divided in relation to midwives’ personal and professional lives.

#### Theme 1. Timeline

Participants described lengths of periods of time that the effects of the event stayed with them and the time they needed to recuperate. These time periods varied in length, between weeks to years. After certain periods of time, the effects of the events wore off although sometimes the memories of the events flared up, caused by similar situations or women that reminded them of the event.

*‘Time heals but it never goes away completely… I will never forget it… there is a scar… up to today.’* (Participant 14)

All participants described that there was a limit to the time period that people around them kept showing their empathy and interest and who continued to offer support and consolation. These time periods did not necessarily match the time that the participant needed to reset and process the experience.

*‘There were limits to their understanding, they moved on, I didn’t.’* (Participant 22)

Some midwives reported that although the event happened a long time ago, they noticed that as they grew older and more experienced, they more often thought back of what had happened at the time, as if ageing and life and practice experience put the event in a different perspective.

*‘I thought it was horrific at the time, but now I’m older and wiser [laughs], I realise how vulnerable we are as midwives and how precious life is.’* (Participant 24)

#### Theme 2. Drawing up the balance of relations with others

For all participants the aftermath of the work-related event served as a landmark, a moment of reflection or as a turning point in relationships with others. This was experienced in professional as well as in personal relationships. Sometimes participants changed their opinion about their colleagues and other (involved) healthcare practitioners after the event. This affected collaborative relationships, sometimes resulting in friction or even dissolution of a midwifery partnership. Opinions about colleagues or other practitioners could change positively or negatively, depending on comments and/or support, resulting in dislike or fondness.

*‘It has really influenced how I now think about that obstetrician, and working with him.’* (Participant 9)

None of the participants had altered her way of relating to women or building or establishing rapport after the event. Midwives did share their experiences of the event with the woman involved but never with other women in their care.

All participants reported that they did not want to burden other women in their care with how they felt, also being afraid that women might lose faith in them because of the event — jeopardising the trusting relationship. Despite these feelings or the fact that they felt stressed or were very aware that management of care differed from that before the event and that they put on a brave face, they all thought this went unnoticed by women.

*‘Women could not have noticed how I felt, no, I just put on a smile, pretending to be fine, no, they will not have noticed how I really felt…I couldn’t allow myself to cry in front of them.’* (Participant 11)

Participants felt enormous impact when they thought that they could have lost a mother or child they were taking care of. It made them feel more protective towards maternal and foetal/neonatal health, even to their own children’s health. In case of maternal, foetal or neonatal loss, midwives experienced feelings of mourning.

*‘It felt if I had lost someone.’* (Participant 21)

Midwives were more likely to share their experiences with midwife friends than with non-midwife friends, because non-midwife friends did not always show the understanding that participants needed, sometimes resulting in arguments and even discontinuation of friendships.

*‘She was just not getting it, saying all the wrong things, making me feel worse, mind you, how could she understand, being a teacher… what is friendship worth, eh?’* (Participant 12).

Sharing experiences with family members, predominantly parents were reported as warm, loving and comforting. Midwives reported that the event served as a parameter for the quality of their partner relationship; either it enhanced the quality when they were able to talk to their partner, or the relationship deteriorated because of being unable to discuss the event with their partner. In one case the aftermath of the event even served as a catalyst for a divorce. Participants were very aware of the impact of the event on their relationships and how it also affected the intimacy with their partner.

*‘I was very aware of the strain it put on the relationship with my husband, I was somewhere else… but we managed and came out stronger at the end.’* (Participant 10)

#### Theme 3. Fretting and worrying

All midwives reported to have become more stressed, tense, insecure, emotional or more easily agitated after the event — at work and at home. All midwives reported an increase in feelings of fear. They worried more about work-related and non-work-related things, sometimes resulting in panic attacks, fatigue and/or loss of sleep.

*‘Worry has become my middle name. Sleeping tablets are my friends.’* (Participant 4)

Participants reported various experiences of emotional lability and instability: feelings of powerlessness, insecurity, loneliness, guilt, fear, worry, anger, stress, sadness, hurt, self-blame, self-pity, doubt, latent anxiety, frustration, moodiness, becoming defensive and apprehensive, feeling scared and having depressive thoughts. They reported experiencing a lack of humour, lack of concentration, physical stress responses, insomnia and having nightmares. Some participants reported constant emotional lability, but most midwives had experienced periodical or intermittent lability; both gradually easing off.

*‘The worst thing is, it doesn’t go away completely, it flares up sometimes but overall eases off, it took ten years.’* (Participant 18)

A similar situation or similar women always reminded midwives of the event, causing a stress response.

*‘I notice when I pass her house or her sister comes for an antenatal visit, I get sweaty palms… feeling uncomfortable….’* (Participant 13)

Some participants described their fear of a similar situation happening again. They described their anticipation when taking a decision, incorporating the possibility of such an event to reduce its recurrence.

*‘Thoughts of “what if” make me do anything to eliminate the possibility for it to happen again.’* (Participant 16)

#### Theme 4. Lessons learned

The events always taught midwives something; about themselves, their management of care or their professional behaviour. They all drew up ‘take-home messages’ from the event and gave meaning to the aftermath of the event. Sometimes the events marked an irreversible point in their career. All midwives described how the events affected their practice and professional behaviour. They all adapted their practice as a result of the event, letting the experience, but predominantly their fears or anticipating decision-regret, rule their decisions and management of care to varying degrees.

*‘Homebirth has never been the same again. It [event] still influences how I act during a homebirth to women… since then. I tense up. I administer Syntocinon at every home birth but not necessarily when the woman births in the hospital.’* (Participant 2)

*‘I think I am less woman-centred than before… less flexible.’* (Participant 23)

*‘I rather have the woman to be dissatisfied with my care than a dead baby.’* (Participant 17)

It even resulted in change of practice (location) or transferring from a community to a hospital setting or vice versa. These changes resulted from knowing that they did not want to continue practising in similar circumstances.

*‘I changed from working in a rural practice to a practice in [name town] because there is a hospital nearby.’* (Participant 3)

All participants reported that the event contributed to or advanced their development of being (or becoming) a reflective practitioner. The event also contributed to personal growth and feelings of transformation.

*‘At the end of the day, it allowed me to grow… I wouldn’t be where I am right now if it [event] wouldn’t have happened… maybe it even made me a better person… a better midwife.’* (Participant 20)

*‘I am proud of myself, I have become a stronger person.’* (Participant 2)

The midwives in our sample were very aware that they had been able to manage emergency or complex situations; skills they had acquired during midwifery education and through lifelong learning that enabled them to deal with complications that had arisen. This was a very reassuring thought to them.

*‘I have learned how to resuscitate… I was full of adrenaline but knew what to do… I did it… I did not doubt my abilities… feeling completely drained and upset afterwards though… .’* (Participant 15)

Some midwives reported that they have learned to listen to their ‘gut’ feeling and to trust this.

*‘I learned to listen to my intuition.’* (Participant 8)

Some midwives reported that the event enhanced their awareness of the importance of being well organised, on an individual, practice and multi-disciplinary level. Some midwives reported that they needed positive experiences to learn again to practice without apprehension and feel comfortable again. New positive experiences helped midwives to gradually recover from the impact of the incidence.

*‘Positive births are healing.’* (Participant 1)

## DISCUSSION

This study investigated the types of response to and perceived impact of work-related traumatic events, including witnessing birth trauma and care-related interpersonal trauma. There was a high degree of similarity in events described by midwives in our study compared with the literature^4-6,14-18^. Findings from our study, however, emphasise the vulnerability of relationships of midwives, on a professional as well as personal level — when involved in a work-related traumatic event. It is known that midwives who are involved in or witness birth trauma, including mismanagement of care and inappropriate interventions, are known to report more severe post-traumatic stress symptoms^4,14-17^. We could not draw any conclusions on the matter as our small sample size did not allow us to compare groups. Instead, our study reported on the experiences and perceptions of the far-reaching, sometimes very painful, effects and impact on the individual professional and personal life of midwives.

The midwives in our study reported more defensive practice manner, like intervening or referring sooner than they would have done previously. Whilst defensive practice of this kind is not necessarily harmful for mothers, it is however associated with the potential for increasing interventions^28,29^. Midwives also reported changing their practice setting, even considering leaving the profession. This is consistent with recent research with Dutch obstetricians where similar responses and actions were consequences of witnessing work-related traumatic events^30^.

Similar to a study by Sheen and collaborators^31^, midwives in our study identified a need to manage personal feelings to maintain a professional appearance; they tried to keep from women in their care how they felt and to what extend the event affected them emotionally and practically, although their management of care and professional behaviour changed after the event. Women, and their individual care, can thus be affected by an event that was completely unrelated to them. This emphasises the fact that the midwife is as much a key player in the care relationship as the woman^3^. The participants reported that debriefing with colleagues, family and friends assisted them in working through their feelings and emotions in the aftermath of the event. The event also aided the participants to learn from their experiences and to use these for professional and personal development. Our participants reported on how they gave meaning to the event. They demonstrated to be able to focus on the positive aspects of an adverse situation, adopting active coping, seeking peer, social and professional support, and adopting a positive self-concept and strategies associated with resilience^32-34^.

Based on our findings, it might be useful to raise (student) midwives’ awareness of the types and impact of work-related traumatic events. Understanding strategies to cope with the impact of such events may assist to alter midwives’ perceptual, cognitive and behavioural responses to work-related traumatic events. Furthermore, continuing professional training for midwives needs to consider midwifery-specific features of work-related trauma exposure such as midwives’ close relationships with women during labour and birth, but also how to handle effects and impact of the event in one’s personal life. Debriefing and counselling may assist midwives who suffer from upsetting, distressing and traumatic work-related experiences. In addition, further research to identify ways of facilitating resilience in midwives following exposure to trauma is warranted.

We used the scores of five items to identify the likelihood of PTSD but did not compare the findings against a structured clinical interview to confirm the likelihood or presence of post-traumatic stress, depression or anxiety amongst our participants^31^. This might be advisable for future research. The majority of people who experience a traumatic event will not develop post-traumatic stress^35^. Our findings of post-traumatic stress acknowledged this and were consistent with an earlier Dutch study^10^. However, it might be useful for future research to compare perceptions of midwives with and without elevated levels of stress following trauma exposure, to identify any differences in experiences, impacts or coping responses. Through this, preventive and supportive strategies can be developed.

A number of limitations are apparent in this study and may affect the usability of its findings. Our study was represented by predominantly Dutch midwives, thus the events that were described occurred mainly within the Dutch maternity context and to some extent within the Flemish context of maternity services. Therefore, our results have limited transferability. Self-selection may have led to selection bias and it is likely our respondents were those for whom the study was most relevant and therefore biased to those with experiences of traumatic work-related events. We are aware that half of the participants in Phase Two had participated in Phase One. As we did not concurrently collect the data in these phases and because we used the same selection criteria in both phases, we believe we did not introduce bias^20^. It is likely that there were midwives we were not able to reach but whose experiences are of importance; those who have never disclosed, those who may have felt they had nothing to offer to this particular study or midwives who have reached closure, not wanting to revisit their experience. There might also have been the possibility that midwives did not wish or were not able to articulate the impact of their traumatic experiences. Consequently, the results may be an underestimate or overestimate of the phenomenon at hand. Retrospective reporting means that perceptions of the nature of the work-related traumatic event and emotions may have been modified over time and therefore might differ from reports obtained immediately following the traumatic event. We did not provide a definition or criteria for what constitutes a work-related traumatic event. Based on the narratives, we believe that midwives had interpreted the word trauma correctly as selfreported types of work-related events included experiences involving actual or threatened death or injury or threat to integrity of self or others — congruent with the definition of a traumatic event^24^.

## CONCLUSIONS

Various work-related traumatic events can impact on midwives’ professional and/or personal life. Accounts of various causes were given, relating to witnessing birth trauma and care-related interpersonal trauma. Midwives experienced a profound effect on day-to-day practice and to a lesser extent on their personal life. Although not all midwives reported experiencing (lasting) effects of the events, the impact on some was far-reaching. Therefore, midwives’ responses to the impact and experiences of workrelated traumatic events cannot be ignored in midwifery practice, education, supervision or mentoring. Further research is certainly warranted.
