# Supplementary material for: Reports of work-related traumatic events: A mixedmethods study
Source: Eur J Midwifery. 2018 Dec 31;2:18. doi: 10.18332/ejm/100611 (PMC7846040; doi:10.18332/ejm/100611)
Supplement: Supplementary file 1 [file EJM-2-18-s1.pdf]

Supplement 1. Questionnaire PHASE 1 (demographic/personal details excluded)

1. **Please describe a work-related incident you have experienced as very upsetting, very distressing or traumatic**………………………………………………………………………………… *(1000 words)*

2. **How did you respond to this event?** (*Multiple answers possible*)
   - I have written down my experiences and feelings (in a diary)
   - Discussed it with family/friends
   - Discussed it with my colleagues
   - Career coaching/supervision
   - Discussed it with the woman (and her partner)
   - Discussed it with my general practitioner
   - Professional help/support
   - Discontinued working as a midwife
   - Nothing
   - Other……………………………………………… *(50 words)*

3. **Does/did the event influence your work on a day-to-day basis?**
   - Not at all
   - Seldom
   - Sometimes
   - Often
   - All the time

4. **When the event does/did influence your work on a day-to-day basis, in what way are you/did you become aware of this?** ....*(500 words)*

5. **Does/did the event influence your personal life?**
   - Not at all
   - Seldom
   - Sometimes
   - Often
   - All the time

6. **When the event influenced your personal life, in what way are you/did you become aware of this?**...*(500 words)*

7. **Does the event still play a distressing role in your life and currently affect your professional, personal or social life?**
   - Not at all
   - A little bit
   - Somewhat
   - Much

- Very much

8. **Does the event still cause intense fear or panic or helplessness?**
   - Not at all
   - A little bit
   - Somewhat
   - Often
   - Very often

9. **Do you currently experience any of these signs/symptoms: irritability, outbursts of anger, lack of concentration, difficulty sleeping, palpitations and/or (excessive) sweating when you think back to what happened?**
   - Not at all
   - A little bit
   - Regularly
   - Often
   - Very often

10. **Do you still have upsetting thoughts, memories, dreams, flashbacks or images that replay what happened?**
    - Not at all
    - Seldom
    - Sometimes
    - Often
    - Very often

11. **Are you deliberately trying <u>not</u> to think about what happened, to avoid places or people related to the event because you feel upset by reminders of the event?**
    - Not at all
    - Seldom
    - Sometimes
    - Often
    - Very often

© 2018 Fontein-Kuipers Y.
